# DIFFERENTIAL ROLES OF COVID-19-RELATED STRESSORS IN MENTAL HEALTH PROBLEMS: A NETWORK APPROACH

**DOI:** 10.1093/geroni/igac059.2985

**Published:** 2022-12-20

**Authors:** Wen Zhang, Tianyin Liu, Dara Kiu, Yi Leung, Wai-wai Kwok, Lesley Sze, Gloria H Y Wong, Terry Lum

**Affiliations:** The University of Hong Kong, Hong Kong, Hong Kong; The University of Hong Kong, Hong Kong, Hong Kong; The University of Hong Kong, Hong Kong, Hong Kong; The University of Hong Kong, Hong Kong, Hong Kong; The University of Hong Kong, Hong Kong, Hong Kong; The University of Hong Kong, Hong Kong, Hong Kong; The University of Hong Kong, Hong Kong, Hong Kong; The University of Hong Kong, Hong Kong, Hong Kong

## Abstract

COVID-19-related stress is heterogeneous and associated with increased mental health conditions in older adults. This study is to investigate relationships between different stressors and how different stressors may increase risks for mental health conditions through a network approach. A telephone survey was conducted among 4,921 older adults (age≥60) from April to June 2022 during the biggest community outbreak of COVID-19 in Hong Kong. The validated 8-item COVID-19-Related Stress Scale (CSS-old) (Cronbach’s α: 0.91) was used to investigate the different stressor for older people in Hong Hong. Respondents were screened for depression using the Patient Health Questionnaire-2 (PHQ-2), anxiety using the Generalized Anxiety Disorder 2-item (GAD-2), and stressors with the CSS-old, 4708 responded to all questions. A regularized partial correlation network via graphical LASSO procedure was computed to analyze the relationship between 8 stressors; a directed acyclic graph (DAG) via a Bayesian hill-climbing algorithm was generated from CSS-old and comorbidity network with PHQ-2 and GAD-2 items. Network analyses identified CSS-old item 6 (families or friends infected), item 3 (daily life interrupted), item 5 (fear of infection affecting the family), and item 8 (worry for community’s health) as the core stressors. DAG analysis found a key triggering role for item 1 (suspension of community services), and the activation of the mental health problems occurred through item 1, which bridged the COVID-19-related stress and mental health problems. These findings suggested that providing support for families with COVID-19 patients and alternative services during community service suspension may reduce mental health problems risks.

